# Supplementary figures and images for: SARS-CoV-2: Possible recombination and emergence of potentially more virulent strains
Source: PLoS One. 2021 May 25;16(5):e0251368. doi: 10.1371/journal.pone.0251368 (PMC8148317; doi:10.1371/journal.pone.0251368)

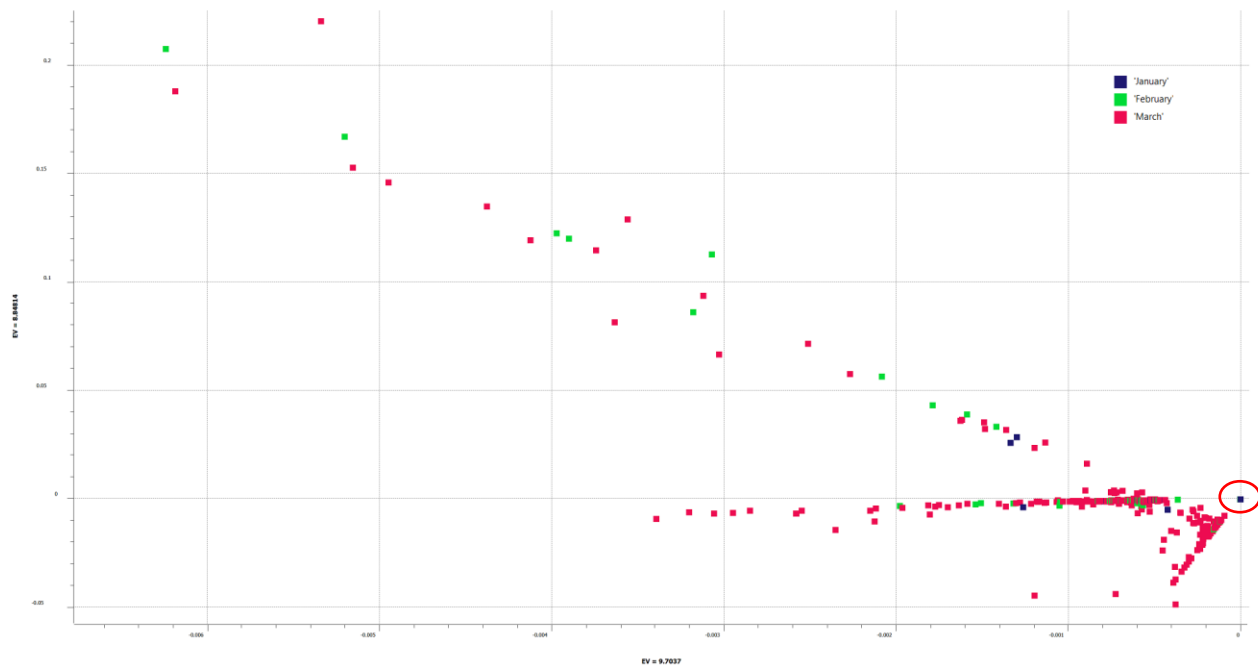

Supplement: S1 Fig — Principal Component Analysis (PCA) blot based on the collection month (January, February, March) for the 2352 SARS-CoV-2 sequences extracted from GISAID data; PC1 (EV = 9.7030), PC2 (EV = 8.84814). Red circle indicates the founder strain that was sequenced in January. (PDF) [file pone.0251368.s001.pdf]

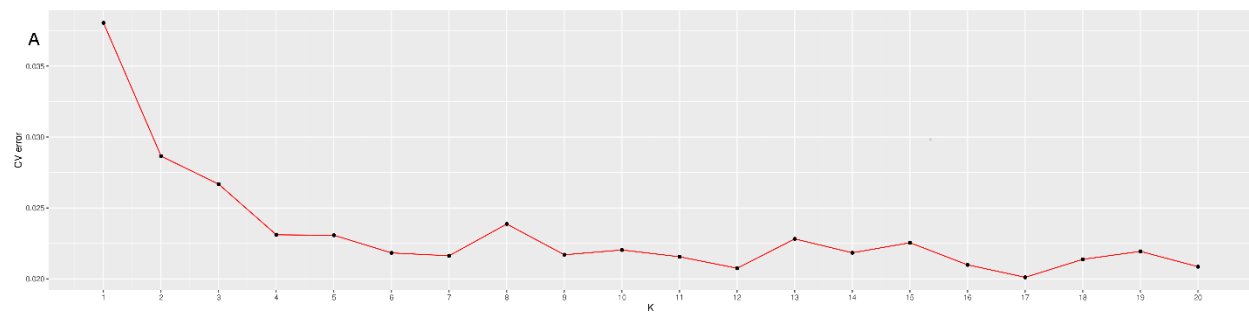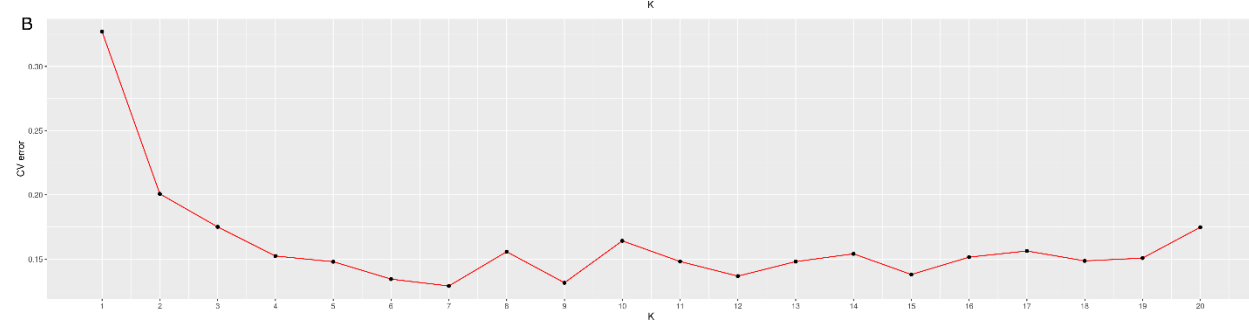

Supplement: S2 Fig — The CV for the MAF>0.5% dataset comprised 72 variants shown. K = 7 is the best fit (upon observing consistency in CV error between the raw set of variants and the set of variants with MAF≥0.5% at K = 7, optimum number of clusters 7 was selected; the inconsistency observed in the raw dataset from K = 8 may be resulting from MAF<0.5% variants), suggesting that 7 different SARS-CoV-2 strains existed in early transmission of SARS-CoV-2 across continents. (CV-cross validation procedure). (PDF) [file pone.0251368.s002.pdf]
