# Supplementary material for: SARS-CoV-2: Possible recombination and emergence of potentially more virulent strains
Source: PLoS One. 2021 May 25;16(5):e0251368. doi: 10.1371/journal.pone.0251368 (PMC8148317; doi:10.1371/journal.pone.0251368)

LD block from combined dataset with MAF≥0.5% set

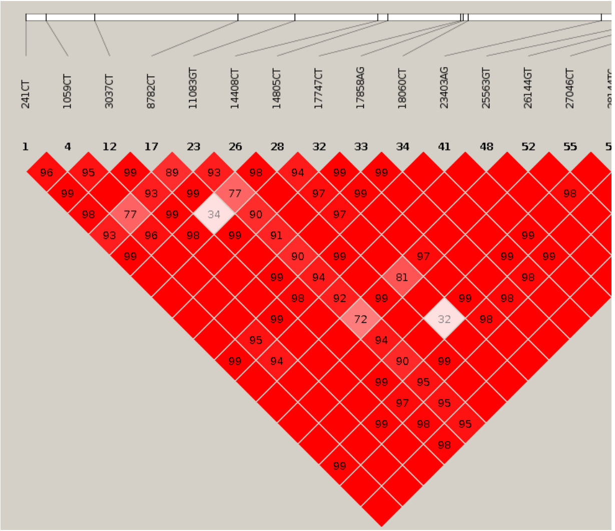

North America

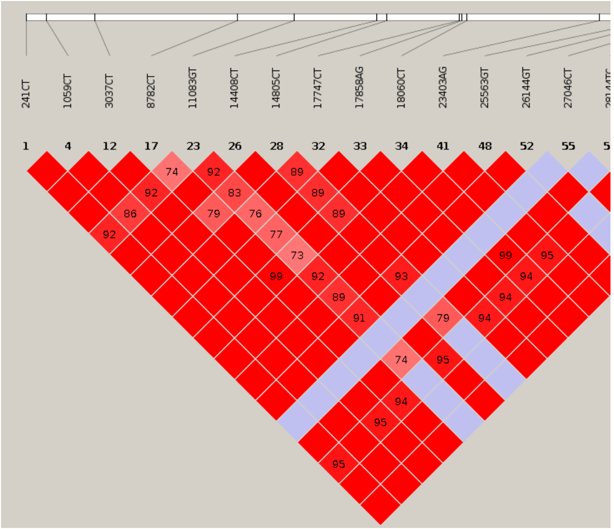

South America

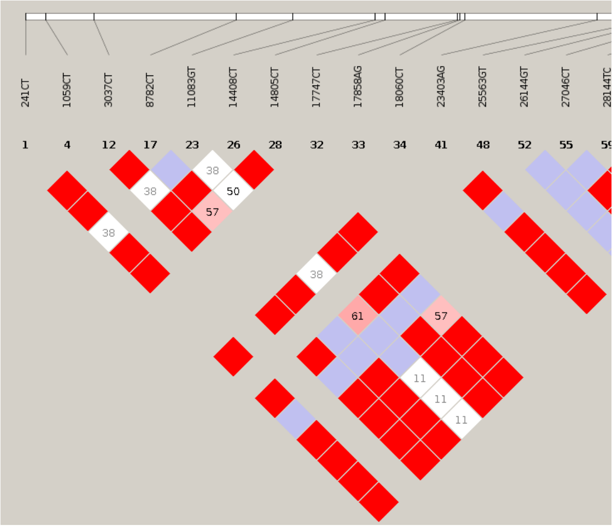

Oceania

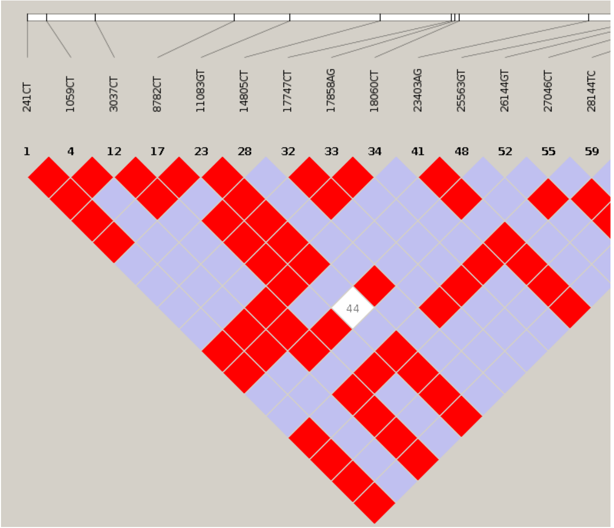

Asia

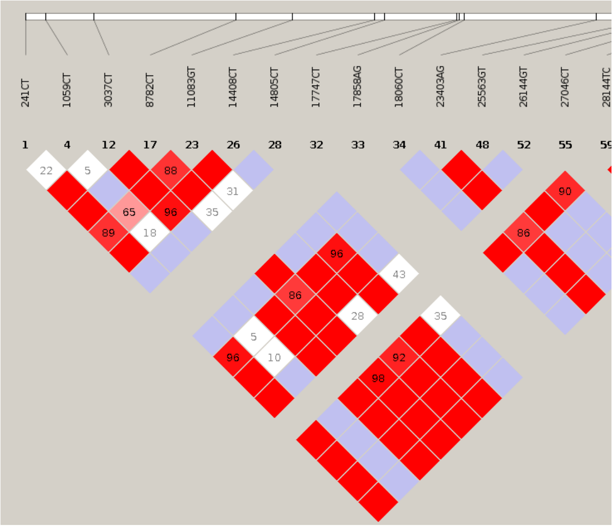

Europe

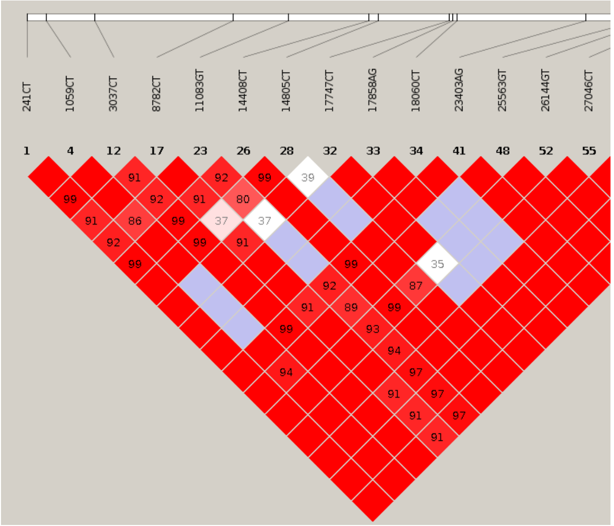

Africa

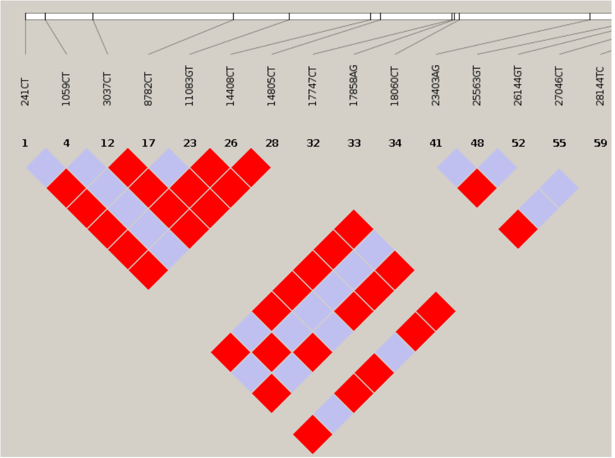

Supplement: S3 Fig — Extent of LD variation observed in each continental dataset when haplotype block comprising the set of 18 variants identified in combined dataset were mapped to continental datasets. (PDF) [file pone.0251368.s003.pdf]
