# Supplementary material for: SARS-CoV-2: Possible recombination and emergence of potentially more virulent strains
Source: PLoS One. 2021 May 25;16(5):e0251368. doi: 10.1371/journal.pone.0251368 (PMC8148317; doi:10.1371/journal.pone.0251368)
Supplement: Attachment — Submitted filename: Response to Reviewers.docx [file pone.0251368.s005.docx]

**PONE-D-20-33456**

**SARS-CoV-2: Proof of recombination between strains and emergence of possibly more virulent ones**

**PLOS ONE**

Dear Dr. Attoui,

We would like to sincerely thank you and the reviewers for your valuable time and useful contributions. We appreciate your input which helped improving our manuscript. **As suggested, we considered all comments that the reviewers made during the revision process specially to improve the presentation of our recombination analyses.** Below you will find our point-by-point response to the referees and the changes we made based on the reviewers’ comments.

**Reviewers’ comments:**

**Reviewer #1:** Hammad and co-workers analyzed early spread SARS-CoV-2 using sequences from GISAID.

The work is important and compelling. They should make a few changes:

Title: “SARS-CoV-2: Proof of recombination between strains and emergence of possibly more virulent ones” should be modified. Suggest: SARS-CoV-2: Possible recombination and emergence of potentially more virulent strains. The analysis does not prove recombination.

We have now changed the title to the one suggested by the reviewer though we now illustrate the possibility by further tools.

The authors should specify the gene for each mutation, at least the first time it is used. For example: Spike_ D614G and Nsp12_P314L.
Specific changes:
Line 34 P314L which is a RdRp mutation likely does not “enhance viral entry and stability.”

We have now changed the sentence and included the genes for each mutation as follows:

“Additionally, we structurally modelled the two most common mutations, Spike_D614G and Nsp12_P314L, which suggested that these linked mutations may enhance viral entry and replication, respectively.”

In our results section we argue that the Spike mutation (D614G) may enhance viral entry and that the RdRp mutation (P314L) may increase replication efficiency. Please see related paragraph and legends.

Line 58 Change Beta-coronaviruses to betacoronaviruses

Change was done as suggested.

Line 220 “cleavage site (Fig 5 C) shows a high surface accessible region, where the viral protein can attach to the host protein.” This should be clarified. The host protein I believe they are discussing is furin (or a furin-like protease). Not clear that this is attachment per se, which is usually reserved for the ACE2 interaction.

Sentence was changed as follows:

“The RRAR cleavage site (Fig 6 C) presents a highly accessible surface region, where the host protease enzyme can readily cleave the S-protein [27]. As such, any mutations on the S protein, close to RRAR furin protease cleavage site, might alter its activity. Therefore, the D614G mutation is believed to increase SARS-CoV-2 virulence [9,32].”

Line 226 and elsewhere change FURIN to furin.

Changes were done throughout the manuscript as recommended.

**Reviewer #2:** The authors claimed that they had proof of recombination between SARS-CoV-2 strains and therefore, the emergence of more virulent ones. The preliminary analysis of recombination is not followed by an in a deep analysis of this process but instead, is filled with another analysis which confounds the reader for the manuscript’s main message.

Regarding the mere analysis of recombination with PhiPack, which implements NSS, MaxChi, and Phi test, the authors said they found evidence of recombination for the European and American datasets.

The evidence of recombination of European recombination only stands for a statistical test, with the rest of the tests largely rejecting the hypothesis. Recombination should be supported by more than one method (see Posada and Crandall 2001; doi:10.1073/pnas.241370698).

For the North America dataset, the recombination signal should be analysed deeper. First, how many permutations did you run At least 100 permutations should be done. It can be seen that MaxChi2 and Phi (with unknown permutations, data not given) almost reject the recombination hypothesis.

All the PhiPack tests were performed using 1000 permutations. It is now clearly mentioned in the manuscript.

We thank the reviewer for the suggestion on performing further tests for recombination. Other than PhiPack methods, we performed additional tests using RDP, GENECONV, MaxChi^2^, Chimaera, and 3Seq from the RDP4. We used the default parameter settings and 1000 permutations. Computationally intensive methods such as Bootscan, SiScan, and LARD were not used.

The results of these additional tests on the North American and European populations are presented in **Table 2.** Possible recombination events in European population is further confirmed with significant p-values by MaxChi^2^, Chimera, and 3Seq tools while possible recombination events in North American populations are confirmed by MaxChi^2^ and 3Seq tools.

| Continental datasets | Event | Start | End | RDP | GENECONV | MaxChi^2^ | Chimera | 3Seq |
| --- | --- | --- | --- | --- | --- | --- | --- | --- |
| North American | 1~ | 530* | 29326 | NS | NS | 3.84E-03 | NS | 1.14E-04 |
|  | 2~ | 226* | 29535 | NS | NS | 1.05E-02 | NS | 2.54E-04 |
|  | 3~ | 175* | 26046 | NS | NS | 1.02E-02 | NS | 4.83E-03 |
| European | 1~ | 820* | 24825* | NS | NS | 9.72E-05 | 2.12E-03 | 4.47E-04 |
| Combined | 1~ | 1519* | 29004 | NS | NS | 1.09E-03 | NS | 1.07E-04 |
|  | 2~ | 29575* | 29835* | NS | 4.98E-05 | NS | NS | 2.56E-02 |

Table 2: Plausible recombination events validated by RDP4 suite. Program did not identify any recombination events in Asian, African, Oceanian, and South American datasets like PhiPack. Analysis was performed using RDP, GENECONV, MaxChi^2^, Chimera and 3Seq algorithms only.

NS=No significant p-value is observed for the recombinant event using respective method.

*= The actual breakpoint position is undetermined (it was most likely overprinted by a subsequent recombination event.

~ = It is possible that this apparent recombination signal could have been caused by an evolutionary process other than recombination.

From Bruen et al., “Max χ2 and NSS falsely infer the presence of recombination under a simple model of mutation rate correlation” (doi:10.1534/genetics.105.048975). Thus, authors need to corroborate further these results. For example, they can try the program ‘profile’ implemented in the PhiPack package, to see with a window approach which regions exhibit the strongest evidence of mosaicism and analysed them.

We further used ‘Profile’ program available in PhiPack suite to identify regions exhibiting strong evidence of recombination with default options for window scan size and step size in all the aligned sequence datasets. Regions showing significant (p<0.05) evidence of recombination is presented in Table 1.

**Table** 1: Results of 4 different tests infer presence or absence of recombination and regions exhibiting evidence of recombination observed from ‘Profile’ program in continental datasets.

| **Dataset** | **Number of informative variants** | **Regions, as identified by Profile, showing significant (p<0.05) evidence of recombination** | **Tests to detect evidence of recombination** | **Significance of observed Phi statistics** |
| --- | --- | --- | --- | --- |
| Africa (n=25) | 19 | 18800-18900, 22450-23425, 29300-29325 | NSS | 1 |
|  |  |  | MaxChi^2^ | 0.978 |
|  |  |  | Phi (permutation) | 1 |
|  |  |  | Phi (normal) | 1 |
| Asia (n=364) | 127 | 4600-4825, 5200-5825, 6425-6500, 6900-7375, 8800-8925, 9175-9750, 9950-10150, 21000-21050, 22650-23625 | NSS | 0.113 |
|  |  |  | MaxChi^2^ | 0 |
|  |  |  | Phi (permutation) | 0.493 |
|  |  |  | Phi (normal) | 0.305 |
| Europe (n=1132) | 276 | 500-700, 2100-2125, 3300-3325, 4050-4725, 5150-5450, 7825-7850, 12925-14075, 18825-18850, 19375-19400, 20075-20475, 22975-23000, 23575-23625, 25275-25325, 26275-26325, 29200-29325 | **NSS** | **0.001** |
|  |  |  | MaxChi^2^ | 0.208 |
|  |  |  | Phi (permutation) | 0.343 |
|  |  |  | Phi (normal) | 0.223 |
| North America (n=738) | 194 | 800-1100, 5575-6300, 6475-6525, 7300-7450, 18800-18875, 19950-19975, 21525-22625, 23500-23600, 24275-24675, 24975-25000, 25725-26050, 26375-26425, 27600-27800, 29300-29325 | **NSS** | **0.007** |
|  |  |  | MaxChi^2^ | 0.061 |
|  |  |  | Phi (permutation) | 0.060 |
|  |  |  | **Phi (normal)** | **0.042** |
| South America (n=24) | 24 | None | NSS | 0.596 |
|  |  |  | MaxChi^2^ | 0.680 |
|  |  |  | Phi (permutation) | 0.717 |
|  |  |  | Phi (normal) | 0.454 |
| Oceania (n=69) | 50 | 22625-23425 | NSS | 1 |
|  |  |  | MaxChi^2^ | 0.502 |
|  |  |  | Phi (permutation) | 0.872 |
|  |  |  | Phi (normal) | 0.361 |
| Combined (n=2352) | 554 | 1625-1650, 3300-3525, 3975-4225, 4625-4725, 5175-5375, 7550-7800, 8800-8925, 9175-9750, 9925-10050, 10600-12125, 13025-14075, 18175-18550, 18850-19150, 20075-20475, 23000-23025, 23475-23525, 24700-24750, 27050-28225 | **NSS** | **0.003** |
|  |  |  | **MaxChi^2^** | **0.001** |
|  |  |  | **Phi (permutation)** | **0.008** |
|  |  |  | **Phi (normal)** | **0.015** |

Which variants would be affected by recombination? Are those variants the ones further analysed in this work?

We have now a new figure (**Figure 5)** depicting regions showing strong LD and regions showing weak LD. Regions of strong LD are supposed not to be broken by recombination. Regions of weak LD are affected by recombination. Variants from such weak LD regions are affected by recombination. As recombination is continental-specific, we think that Figure 5 clearly explains regions/variants affected by recombination. For the genetic substructure analysis, we used all variants including both the strong LD and weak LD variants.

Other methods can be also included. For instance, the authors determine the linkage disequilibrium (LD) but they did not correlate with the genetic distance, which is the test for recombination.

As mentioned above, we have now performed correlation between LD and genetic distance and have illustrated continental-specific strong LD and weak LD regions.

Figure 5: Correlation between LD and physical distance across continental SARS-nCoV-2 genomes. **(A)** Upward peaks show strong LD and downward peaks show weak LD; North America and Oceania show strong LD in the region overlapping the S protein (hypothetically overlapped on its genome structure); strong LD among variants suggests that these de novo mutations were not broken by events of recombination **(B)** Smooth lines show clear differences in LD over physical distance across continental data sets, **(C)** The genome structure of SARS-CoV-2 is depicted.

As the authors do not provide enough evidence of recombination, the message of this work should be changed.

Though we have now performed additional tests for recombination suggested by the reviewer, we have changed the title of the manuscript following the suggestion of Reviewer 1. The new title is: “**SARS-CoV-2: Possible recombination and emergence of potentially more virulent strains”.**

Finally, alignments use for test recombination should be made accessible in case authors proved that.

Alignments used for tests of recombination are listed in the Supplementary File 1.

Finally, we would like to thank you again and we look forward to hearing from you regarding our submission. We would be glad to respond to any further questions and comments that you or the reviewers may have.

Sincerely yours,

**Dr. Fahd Al-Mulla**
Genetics and Bioinformatics Department

Dasman Diabetes Center, Kuwait
[fahd.almulla@dasmaninstitute.org](mailto:fahd.almulla@dasmaninstitute.org) | [www.dasmaninstitute.org](http://www.dasmaninstitute.org)
